# Academic treatment optimization studies in oncology and internal medicine: a risk- and burden-adapted regulatory framework for the European Union

**DOI:** 10.1016/j.lanepe.2026.101888

**Published:** 2026-10-15

**Authors:** Wolfgang E. Berdel, Martin Dreyling, Martin Schrappe, Christoph Wanner, Georg Schmidt

**Affiliations:** a Department of Medicine A, University Hospital Münster, Albert Schweitzer Campus 1, Münster 48149, Germany; b Department of Medicine III, Klinkum Großhadern, Ludwig Maximilians University, Marchionini Street 15, München 81377, Germany; c Pediatrics I, University Hospital Schleswig-Holstein, Arnold Heller Str. 3, Kiel 24105, Germany; d Department of Clinical Studies and Epidemiology, Comprehensive Heart Failure Center, University Hospital Würzburg, Am Schwarzenberg 15, Würzburg 97078, Germany; e Department of Medicine I, TUM Universitätsklinikum Rechts der Isar, Technische Universität München, Ismaningerstr. 22, Munich 81675, Germany

**Keywords:** Treatment optimization trials (TOS), EU clinical trials regulation (CTR), EU Biotech Act

## Abstract

Academic treatment optimization studies (TOS) play an important role in determining how established and emerging treatments should be combined, sequenced, intensified, de-intensified, or otherwise optimized in routine clinical care. Unlike commercial clinical trials, which primarily support the development and regulatory approval of new medicinal products, academic TOS frequently address questions for which commercial incentives are limited. Such studies therefore contribute directly to the evidence base underlying clinical guidelines and health-care standards. A substantial proportion of academic TOS involves authorized medicinal products used outside the strict wording of their marketing authorization (label), but within evidence-supported clinical practice. Current European clinical-trial regulation does not always adequately reflect the distinction between the label of a medicinal product and the incremental risk and burden introduced by a particular treatment optimization strategy. Increasing administrative and regulatory requirements disproportionately affect investigator-initiated multicentre studies with limited resources. This article presents a policy perspective derived from the experience of investigators, clinical researchers and ethics committee members involved in academic clinical trials. We propose a risk- and burden-adapted regulatory approach for eligible academic TOS. The relevant reference point for assessing proportionality should be normal clinical care rather than the label alone. Evidence-supported TOS involving authorized medicinal products, particularly studies corresponding to OECD categories A and B1, should be considered for a risk-proportionate regulatory pathway when the proposed intervention introduces minimal additional risk and burden compared with normal clinical care. Selected OECD B2 studies could potentially qualify where additional evidence and expert assessment demonstrate similarly low incremental risk. The assessment should be performed on a trial-specific basis. Relevant standards of routine care should be defined by the respective medical societies in the field. Ethics committees, supported by physicians experienced in the relevant disease area, should assess incremental risk and burden. Monitoring, oversight and reporting requirements should likewise be risk-proportionate and prospectively justified in the trial protocol. The objective is not deregulation of academic clinical research but risk-proportionate regulation. A risk-adapted framework for academic TOS could strengthen investigator-initiated evidence generation, particularly for questions with limited commercial incentives, and improve the evidence base for optimizing routine clinical care in Europe.

## Introduction

The development of effective systemic cancer therapies started after WW II,^1^, ^2^, ^3^, ^4^ it has been a stepwise process in which established treatment modalities have repeatedly been combined,^5^ sequenced, intensified, or de-escalated. Randomized clinical trials^6^ have been central to this process, not only for evaluating novel therapies but also for determining how established interventions can be optimally integrated into clinical care. This latter function has been particularly important in oncology, where cooperative academic study groups established successive generations of randomized treatment optimization studies (TOS) comparing new treatment strategies with established standards of care.^7^

TOS address questions that are distinct from, although complementary to, the development of new medicinal products. Commercial clinical trials sponsored by the pharmaceutical industry primarily seek to establish the efficacy and safety of investigational medicinal products for regulatory approval and subsequent market access. Academic TOS, in contrast, frequently investigate how interventions that are already available in clinical practice should be used. Questions include the optimal dose, duration and sequencing of treatment, the combination of systemic, surgical and radiotherapeutic modalities, treatment intensification or de-intensification, and risk-adapted treatment according to predefined patient characteristics. Such questions are directly relevant to the effectiveness, safety, efficiency and sustainability of healthcare delivery, but may provide limited commercial incentives for their systematic investigation. Academic studies therefore address important areas of evidence generation that would otherwise remain insufficiently investigated, including rare diseases, vulnerable patient populations, treatment de-escalation and comparative optimization of established therapies.

The importance of TOS is particularly apparent as the number of effective therapeutic options increases. In oncology, successive generations of randomized academic trials have contributed to the development of multimodal treatment strategies and to improvements in survival and treatment tolerability. Pediatric oncology provides a particularly illustrative example: across major childhood malignancies, event-free survival increased substantially over successive generations of treatment optimization studies, from below 20% in the 1950s to more than 60% and, for some diseases, approximately 90% by the early 2000s (Fig. 1).^8^ These improvements cannot simply be attributed to treatment change alone, as they occurred alongside advances in diagnostics, supportive care, risk stratification and multimodal treatment. Nevertheless, many of these theoretic confounders were prospectively incorporated into trial strategies, again underlining the role of academic TOS in translating advances in treatment into standards of care.

**Fig. 1:**
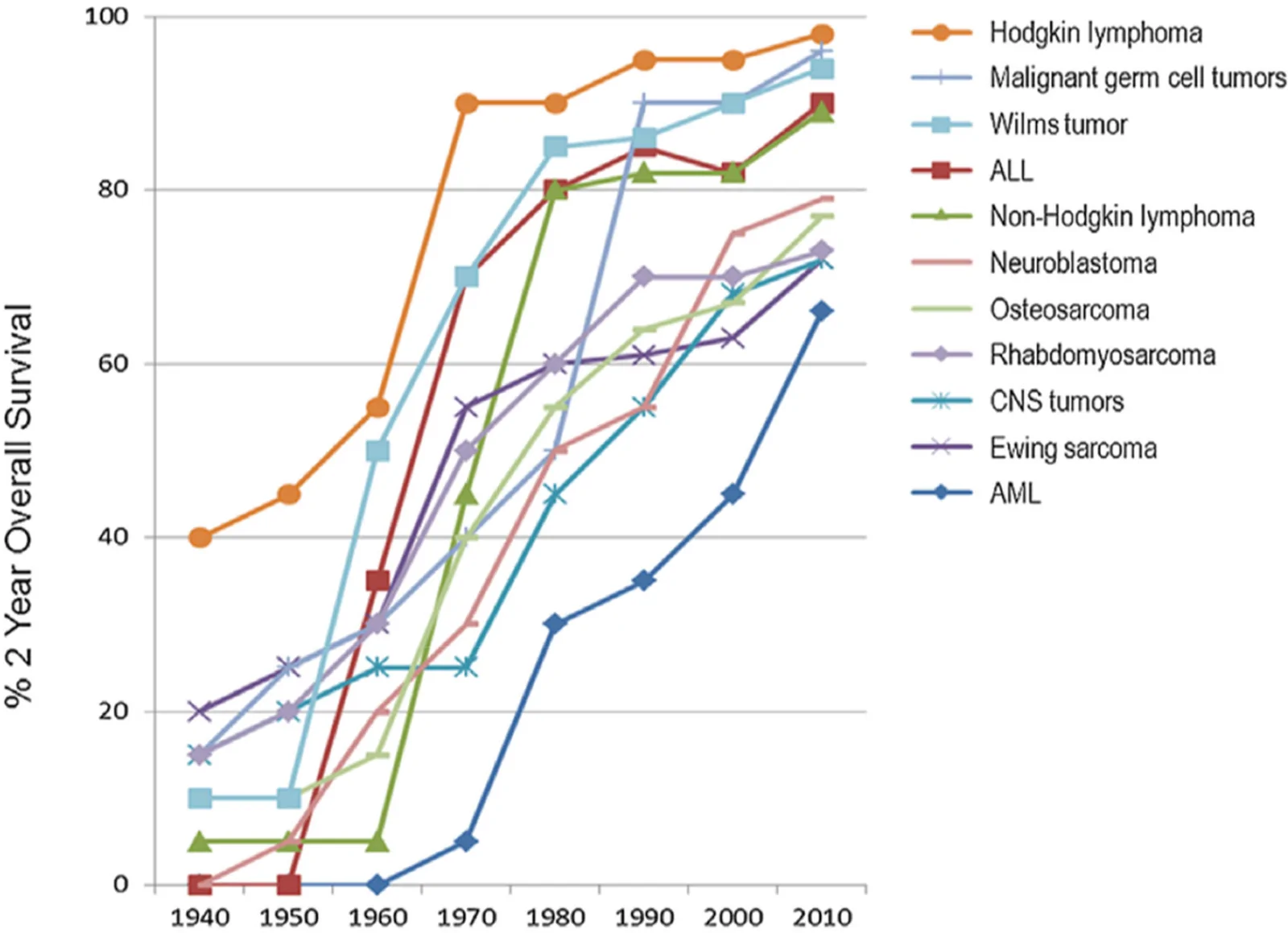
Improvement of cancer survival rates in children in Germany between 1940 and 2010. Reproduced with modification from Rossig C et al.^8^ with permission of Wiley Periodicals Ltd.

Treatment optimization also became an important component of adult oncology. Across hematological and solid malignancies—including leukemia, lymphoma, germ-cell tumors, breast cancer, colorectal cancer and lung cancer—academic randomized trials have addressed the integration, sequencing, duration and intensity of multimodal treatments. The development of adjuvant breast cancer therapy provides a prominent example of how investigator-initiated randomized research could establish treatment strategies that subsequently became standards of care and saved the lifes of many patients.^9^ Similar optimization questions have emerged across internal medicine. In cardiology, nephrology and other fields, multiple investigator-initiated randomized trials have established how combinations of established treatments should be sequenced, intensified or otherwise optimized. Due to space limitations only two such studies are quoted here.^10^^,^^11^

A substantial proportion of academic TOS investigates authorized medicinal products in treatment settings that extend beyond the wording of the marketing authorization. The OECD classification^12^ distinguishes clinical trials according to the regulatory status and clinical use of medicinal products, including category A for authorized, in-label use, B1 for authorized medicines used off-label but supported by published evidence, guidance or established medical practice, B2 for off-label use without such support, and C for non-authorized products. Many academic TOS fall within categories B1 and, less frequently, B2. Importantly, however, the regulatory status of a medicinal product does not necessarily correspond to the incremental risk introduced by a particular treatment optimization question. An evidence-supported change in dose, duration, sequence or combination of established treatments may involve little additional risk compared with routine care, even when the specific use is outside the wording of the marketing authorization. This distinction is central to the policy problem addressed in this paper.

## Regulatory challenge for academic treatment optimization

The International Council for Harmonisation of Technical Requirements for Pharmaceuticals for Human Use (ICH) was established to promote harmonization of technical requirements for the development and registration of medicinal products. Its Good Clinical Practice (GCP) guideline subsequently became an important international reference point for the conduct of clinical trials and was incorporated into the European regulatory framework.

Implementation of the EU Clinical Trials Directive CTD 2001/20 introduced additional requirements for clinical trial authorization, sponsor responsibilities, monitoring, safety reporting and documentation. For investigator-initiated studies with limited infrastructure and funding, these requirements represented a substantial additional administrative and financial burden. Academic TOS were particularly vulnerable because they frequently required multicentre infrastructure without the commercial background available to industry-sponsored drug development.

Consequences for academic treatment optimization research again are best illustrated by pediatric oncology in Germany. Klingebiel et al. reported that the number of open TOS conducted by the German Society for Paediatric Oncology and Haematology decreased from 33 in 2002 to 2 in 2017.^13^ During the same period, the number of drug trials under CTD 2001/20 increased and the number of clinical registries increased substantially.^13^ Fig. 2 illustrates a temporal association between these developments and the implementation of CTD 2001/20. Although these observations do not establish causality, they are consistent with concerns regarding the increasing administrative and regulatory burden for academic drug trials. They do illustrate a marked shift in the structure of academic clinical research during the period following implementation of CTD 2001/20. Disease-specific registries became increasingly important as reference structures for standardized patient care, quality assurance and data collection. Registries can document real-world care and supporting quality improvement, but they cannot replace randomized prospective studies when comparative evidence on treatment strategies is required and innovation is an objective.

**Fig. 2:**
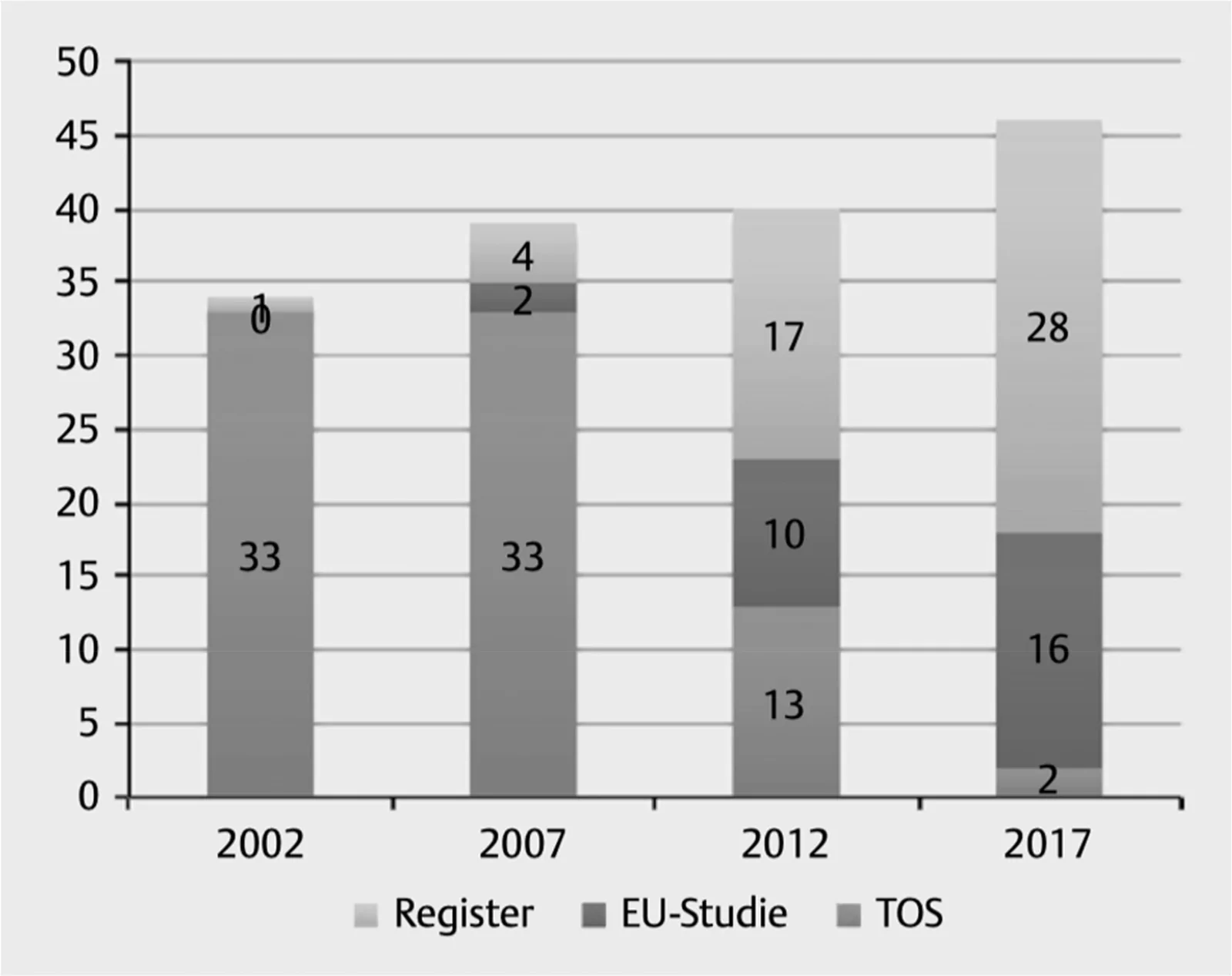
Change of numbers in study types over time. Survey of the Society for Pediatric Oncology and Hematology (GPOH); In 2002 33 TOS of GPOH were open. This number went down to 2 in 2017. Over the same period of time drug studies under CTD 2001/20 (EU-Studie) increased from 0 to 16 and disease-oriented registries from 1 to 28. Registries often replaced former TOS. Reproduced from Klingebiel T et al.^13^ with permission of Thieme-Verlag.

Illustrative data from the experience of one of the authors regarding trial-specific costs and timelines before and after implementation of the CTD 2001/20 are presented in Table 1. These data are intended as an illustrative case rather than as a systematic assessment of regulatory costs. They nevertheless illustrate the substantial resources that are required to initiate and conduct academic drug trials under the regulatory framework.

**Table 1:** Increase of costs, time and paperwork for academic studies (all phases) before and under different EU regulations.^a^

Hurdles	Phase/center	Before CTD 2001/20 (<2004)	Under CTD 2001/20 and CTR 536/2014 (>2004)
Time	Phase I monocenter^b^	1 month^c^	>1.5 years^c^
	Phase I monocenter^d^	2 years	>4 years
	Phase II/III multicenter^b^	3 months	>2 years
Costs	Phase I monocenter	approx. 30,000€	>200,000€^c^
	Phase II/III multicenter	<50,000€	approx. 500,000€
Paper	Phase I monocenter	approx. 100 Pages	>3000 Pages
	Phase II/III multicenter	approx. 200 Pages	>3000 Pages

a Personal approximations for studies conducted by one of the authors (WEB); FPFV, first patient first visit.

b From protocol to FPFV.

c Experience of the author.

d From protocol to publication.

The question is whether similar changes occurred beyond oncology. Data collected by the Ethics Committee of Westphalia-Lippe, Germany, between 2006 and 2012 provide a further illustration. During this period, the number of commercial drug trials decreased only from 38 to 29, whereas investigator-initiated academic drug trials declined from 6 to 2. At the same time, the number of registries increased from 1 to 11. Although these descriptive data cannot establish simple causality, the concurrent decline in investigator-initiated interventional studies and increase in registry-based activities are consistent with a shift towards observational approaches during a period of increasing regulatory requirements, which was also reported from other EU member states.^14^

The interpretation of trends in investigator-initiated trials is nevertheless complicated by increasing collaboration between academia and industry. Pharmaceutical companies may support investigator-initiated studies when the results are relevant to regulatory approval, label expansion or post-marketing evidence generation. Such collaborations can be highly valuable, but they do not necessarily replace independently initiated studies addressing questions without commercial incentives.

## From CTD 2001/20 to CTR 536/2014

Concerns about declining clinical trial activity and increasing costs contributed to the development of Regulation (EU) No. 536/2014, the Clinical Trials Regulation (CTR).^15^^,^^16^ The CTR introduced important improvements, including a harmonized EU-wide framework, a single submission portal through the Clinical Trials Information System (CTIS), coordinated assessment and increased transparency of clinical trial information. Since 31 January 2025, all clinical trials in the EU/EEA must be conducted under the CTR framework using CTIS.

These changes substantially improved the European clinical trial infrastructure. The CTR provides a common framework for multinational trials and has the potential to facilitate their conduct across Member States. However, harmonization does not necessarily imply risk-proportionality. Investigator-initiated multicentre studies still face substantial administrative hurdles, particularly when complex treatment strategies, multiple interventions or off-label use of authorized medicines are involved. The provisions for these trials are fragmented in different parts of the CTR text and hard to follow by investigators.

The policy question is therefore not whether clinical research should be de-regulated, but whether regulatory requirements are proportionate to the incremental risks and burdens posed by different types of research.

Recent analyses have continued to identify challenges for academic and pragmatic trials, including the administrative requirements associated with complex trial designs and the practical implementation of low-intervention provisions.^17^, ^18^, ^19^ More broadly, European policy analyses have raised concerns about the competitiveness of the EU as a location for clinical research.^20^^,^^21^ These developments suggest that regulatory harmonization alone may not be sufficient to ensure an efficient environment for investigator-initiated evidence generation.

The relevance of treatment optimization is not limited to oncology. In nephrology, for example, the increasing availability of disease-modifying therapies has created new questions regarding their optimal combination, sequencing and intensity, particularly in patients with rare kidney diseases. Academic clinical trial networks are being developed to address these questions, illustrating the continuing need for efficient investigator-initiated research infrastructure.

## Risk-proportionate regulation as policy opportunity

The current European policy environment provides an opportunity to reconsider the regulatory framework for academic treatment optimization studies. In December 2025, the European Commission presented a proposal for a European Biotech Act that includes measures intended to simplify clinical-trial procedures, reduce administrative burden and accelerate authorization processes. The proposal includes amendments to the CTR and introduces further risk-based differentiation of clinical trials, including provisions concerning minimal- and low-intervention clinical trials. The proposed reforms start with a reduction of documents to be submitted for Clinical Trial Application and further represent important steps towards risk-proportionate regulation.

However, an important policy question remains: whether the proposed categories adequately capture academic TOS in which authorized medicines are used outside the strict wording of the marketing authorization but within evidence-supported clinical practice. For such studies, the relevant reference point for assessing additional risk and burden should be established normal clinical care rather than the label alone. Established clinical care should be defined mainly by medical societies in the field using current evidence, professional guidelines, accepted clinical practice and, where appropriate, previous randomized treatment optimization studies.

The following considerations are intended to stimulate discussion on proportionality in academic clinical-trial regulation and should be viewed as a policy proposal derived from practical experience rather than as a formal comparative analysis of regulatory systems. We propose that evidence-supported academic TOS involving authorized medicinal products, particularly studies corresponding to OECD category A and B1, should be eligible for a proportionate regulatory pathway when the proposed intervention introduces minimal additional risk and burden compared with established clinical care. Selected B2 studies might be considered under such a framework only where additional evidence and expert assessment demonstrate that the incremental risk remains minimal. These trials should be further registered through the entry portal CTIS to exploit all its advantages, but then assessed by an ethics committee and not necessarily by a national competent authority. The distinction between regulatory classification and actual incremental risk is important. An intervention may technically constitute off-label use while being well established in clinical practice and supported by substantial evidence. Conversely, an intervention that is formally within an authorization may still introduce meaningful additional risks when used in a substantially different treatment context. Regulatory proportionality should therefore be based on the characteristics of the specific research intervention and its incremental risk and burden rather than on authorization status alone.

## Role of ethics committees and medical societies

The assessment of incremental risk and burden should be performed on a trial-specific basis using predefined criteria and appropriate clinical expertise. Ethics committees should play a central role in this assessment, with participation of physicians experienced in the relevant disease area. The authors recognize that different organizational solutions are possible. The intention of this proposal is therefore not to prescribe a single regulatory architecture, but to encourage discussion of how disease-specific expertise can be incorporated more systematically into the assessment of incremental risk and burden. This would not completely transfer responsibility for participant protection from competent authorities to ethics committees. Rather, it would ensure that assessment of risk proportionality incorporates clinical expertise concerning the actual risks of modifying established treatment strategies.

The definition of established normal clinical care should not be left exclusively to individual investigators. Rather, it could be supported by evidence-based professional guidelines, consensus statements, established treatment protocols and previous randomized TOS. Medical societies In the field should be in charge to defining the clinical reference standards within specific disease areas. Taken the European Society for Medical Oncology (ESMO) as an example they have reached agreement on common guidelines for diagnosis and treatment for most cancer entities and regularly keep them up to date. This approach would also allow the assessment of treatment optimization studies to remain dynamic. As evidence accumulates and treatment strategies become established, the regulatory assessment of subsequent optimization questions could reflect the evolving standard of care.

## Risk-adapted monitoring and oversight

We further propose a risk-adapted approach to monitoring, oversight and reporting, drawing on proposals developed by the Danish Medicines Agency.^22^ The Danish guidance is cited as an example of an existing European risk-adapted approach, not as proof of superiority of a particular regulatory model. The intensity of monitoring, adverse-event reporting and other oversight activities should be proportionate to the anticipated incremental risk and burden of the study and every monitoring and reporting requirements should be justified in the protocol. This would allow the ethics committee to assess whether the proposed measures are proportionate to the actual risks of the study. A risk-adapted framework does not mean less protection for participants, rather, it should mean that resources devoted to monitoring and oversight are directed towards risks that are relevant to participants and data integrity. Such an approach could be particularly relevant for academic multicentre trials in which extensive monitoring requirements can consume a substantial proportion of limited research resources without necessarily providing equivalent additional protection for participants.

## Discussion

Academic TOS represent an essential component of evidence-based medicine. Their purpose is not primarily to establish the market authorization of new medicinal products, but to determine how available treatments can best be combined and used in patients. As treatment options become increasingly complex, the importance of such comparative and optimization research is likely to increase rather than decrease.

This article should be interpreted as a policy perspective rather than a formal policy-analysis study. The proposals arise from the practical experience of investigators, clinical trial leaders and ethics committee members and are intended to contribute to the ongoing discussion of proportionality in European clinical-trial regulation. The regulatory challenge arises because the formal regulatory status of a medicinal product does not always correspond to the actual incremental risk posed by a particular research question. This is especially relevant for evidence-supported off-label use of authorized medicines, but does not imply that off-label trials should automatically be considered low risk. Rather, it suggests that off-label status alone should not determine the regulatory burden, but the expert estimation of additional risk and burden.

This principle is consistent with the broader movement towards risk-proportionate regulation in Europe. The current European Biotech Act proposal explicitly seeks to further differentiate clinical trials according to their risk profile and to adapt regulatory requirements accordingly, also by introducing a minimal intervention clinical trial category for OECD A category trials. Our proposal extends this principle to the specific context of academic treatment optimization. In particular, it emphasizes the importance of considering evidence-supported use of authorized medicines, including OECD B1 and selected OECD B2 studies, when determining whether a trial can be conducted under a risk-proportionate regulatory pathway.

This approach would preserve important safeguards. Participant informed consent and Ethics review would remain mandatory. Clinical expertise would be incorporated into the assessment of incremental risk, rather than replacing regulatory or ethical oversight. A further advantage would be greater transparency of the reasoning underlying regulatory requirements. If the protocol explicitly states why particular monitoring, adverse-event reporting, data verification and other oversight measures are required—or why they are not required to the same extent—the resulting decision becomes more transparent and reproducible.

There are also potential limitations and challenges. First, determining what constitutes established clinical care may differ between countries. Second, professional guidelines may lag behind rapidly evolving evidence. Third, different ethics committees may initially apply different thresholds when assessing minimal additional risk and burden. These challenges argue for the development of transparent criteria and shared guidance rather than for abandoning risk-adapted assessment.

## Conclusions

The development of modern medicine depends not only on discovering and authorizing new drugs but also on determining how existing and emerging treatments should be optimally combined, sequenced, intensified or de-escalated. Academic TOS have made an important contribution to this process across oncology and internal medicine. The European regulatory framework has evolved substantially, and the CTR has created important advantages through harmonization, CTIS and increased transparency. Nevertheless, the continued development of academic treatment optimization requires further consideration of regulatory proportionality.

We suggest that future reforms of the European clinical-trial framework place greater emphasis on incremental risk and burden relative to established clinical care. Evidence-supported TOS involving authorized medicines, particularly OECD category A and B1 studies, should be considered for a proportionate regulatory pathway where the incremental risk and burden are minimal. Selected B2 studies could potentially be considered where additional evidence and expert assessment support a similarly low-risk classification. Monitoring, oversight and reporting should likewise be proportionate to the actual risks of the study and justified prospectively within the trial protocol. Ethics committees, supported by appropriate clinical expertise, should play the central role in assessing trials of these categories. As widely agreed upon^23^ the objective is not deregulation of academic clinical research, but risk-proportionate regulation.

Future development of the European clinical trial framework should involve all stakeholders involved in the generation, assessment and use of clinical evidence, including investigators, patients, healthcare professionals, ethics committees, regulators and policymakers. A risk-adapted framework for academic treatment optimization studies summarized in one specific part of the CTR could strengthen investigator-initiated evidence generation and improve the evidence base for optimizing routine clinical care across Europe.

## Contributors

All authors contributed equally to this manuscript. The views expressed in this article are the personal views of the authors and may not be understood or quoted as being made on behalf of or reflecting the position of their affiliations.

## Declaration of interests

**WEB** is chairperson of the Ethics Committee Westphalia Lippe, board member of the Assoc. of Medical Ethics Committees in Germany and of the European Assoc. of Medical Research Ethics Committees MedEthicsEU. He is co-founder of 2 Biotech Companies Anturec Pharmaceuticals (anturec.com) and Elvesca Biotherapeutics (elvesca.com) and co-inventor of patents of both companies and he is scientific advisor of Philogen S.p.A. (philogen.com).

**MD** is chairperson of the Coalition of Medical Societies in Europe for Reduction of Bureaucracy in Clinical Trials (https://bureaucracyincts.eu). He reports (institutional) support of academic studies from AbbVie, Gilead/Kite, Janssen, Lilly, and Roche; honoraria for independent academic presentation from AbbVie, AstraZeneca, BeOne, Bristol-Myers Squibb, Gilead/Kite, Janssen, Lilly, and Swedish Orphan Biovitrum; congress travel support from Janssen and Roche; and participation on a scientific advisory board for AbbVie, AstraZeneca, AvenCell, BeOne, Bristol-Myers Squibb, Genmab, Gilead/Kite, Incyte, Janssen, Lilly, Novartis, Roche, and Swedish Orphan Biovitrum.

**MSch** is emeritus chairperson of the International BFM group (I-BFM). He is member of the Ethics Committee of the Medical Faculty of the Christian-Albrechts University Kiel. He reports honoraria and research funding by JazzPharma, Servier, and Amgen.

**CW** is board member of the Coalition of Medical Societies in Europe for Reduction of Bureaucracy in Clinical Trials. He received honoraria for steering committee membership, advisory board membership and lecturing from Alexion, Amgen, AstraZeneca, Bayer, Boehringer Ingelheim, CSL-Vifor, GSK, Lilly, Novartis, Novo Nordisk, Pfizer, Sanofi, and Vera.

**GSch** is chairperson of the Ethics Committee of the Technische Universität Munich, and chairperson of the Assoc. of Medical Ethics Committees in Germany. He declares no conflicts of interest.

Final approval was obtained from all authors. All authors accept responsibility for the publication. This work was not funded.
